# Cyclic Thiosulfinates: Synthesis and Applications in Chemical Biology and Materials Science

**DOI:** 10.1002/cbic.70505

**Published:** 2026-08-21

**Authors:** Benjamin Liebson, Jeffrey N. Agar, Roman Manetsch, Prakash T. Parvatkar

**Affiliations:** ^1^ Department of Chemistry and Chemical Biology Northeastern University Boston Massachusetts USA; ^2^ Department of Pharmaceutical Sciences Northeastern University Boston Massachusetts USA; ^3^ Barnett Institute of Chemical and Biological Analysis Northeastern University Boston Massachusetts USA; ^4^ Center for Drug Discovery Northeastern University Boston Massachusetts USA

**Keywords:** chemical probe, oxidation, polymer networks, protein modification, redox biology, sulfur heterocycle, therapeutics

## Abstract

Cyclic thiosulfinates (CTs) constitute a distinct class of organosulfur compounds characterized by a polarized ─S(═O)─S─ motif and inherent ring strain, which together dictate their unique reactivity toward thiols. Recent advances in synthetic methodologies, particularly the controlled oxidation of cyclic disulfides, have enabled access to stable CTs with tunable ring sizes and substitution patterns. Mechanistic studies reveal that ring‐induced hyperconjugation and conformational constraints enhance nucleophilic substitution, allowing for selective cross‐linking of proximal thiols while minimizing the modification of isolated thiols. These properties have positioned CTs as valuable tools in chemical biology for selective thiol targeting and protein cross‐linking in complex biological environments. Concurrently, CTs are gaining prominence as dynamic covalent cross‐linkers in materials science. This review highlights recent progress in the synthesis, reactivity, and applications of CTs across chemical biology and materials science.

## Graphical abstract

Cyclic thiosulfinates (CTs) are strained organosulfur heterocyclic compounds with a polarized ─S(═O)─S─ bond that enables selective thiol reactivity. Advances in synthesis afford tunable scaffolds that support efficient proximal thiol cross‐linking and enable applications in chemical biology and materials science.

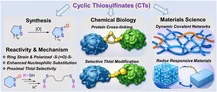

**Abbreviations**
3D: three‐dimensional
ALS: amyotrophic lateral sclerosis
Cbz: benzyloxycarbonyl
CHP: cumene hydroperoxide
CPO: chloroperoxidase
CT: cyclic thiosulfinate
Cys: cysteine
DFT: density functional theory
DMAP: 4‐(dimethylamino)pyridine
DMD: 2,2‐dimethyldioxirane
DNA: deoxyribonucleic acid
DSF: differential scanning fluorimetry
fALS: familial amyotrophic lateral sclerosis
HIF‐1: hypoxia‐inducible factor 1
HPLC: high‐performance liquid chromatography
hPSC: human pluripotent stem cell
L‐DET: L‐diethyl tartrate
*m*‐CPBA: *meta*‐chloroperbenzoic acid
NMR: nuclear magnetic resonance
PEG: polyethylene glycol
SOD1: superoxide dismutase 1
TBHP: *tert*‐butyl hydroperoxide
TMSCl: chlorotrimethylsilane.

## 1 Introduction

Organosulfur compounds play a central role in synthetic chemistry, redox biology, and functional materials owing to sulfur’s unique physicochemical properties, including high polarizability, multiple accessible oxidation states, and capacity for reversible covalent bond formation [1, 2, 3, 4, 5]. Among these, thiosulfinates (R─S(═O)─S─R′) constitute a unique class of organosulfur compounds, characterized by the oxidation of one sulfur atom in a disulfide (R─S─S─R′) to the sulfoxide oxidation state. This transformation generates a highly polarized ─S(═O)─S─ linkage, which imparts pronounced electrophilicity to the sulfenyl sulfur atom and equips thiosulfinates with a reactivity that is distinct from those of their disulfide precursors [6, 7]. While thiosulfinates have been identified as oxidation products of disulfides for several decades, their functional potential has only recently been explored.

The foundational understanding of thiosulfinate chemistry originated from mid‐20th century investigations into sulfur‐containing natural products [8, 9, 10] and the degradation pathways of penicillin [11, 12, 13]. Historically, scientific interest in thiosulfinates has been focused on naturally occurring acyclic derivatives such as allicin, which is enzymatically produced from garlic (*Allium sativum*) [14, 15, 16, 17]. Allicin and structurally related *S*‐oxide disulfides demonstrate notable antimicrobial, antioxidant, and thiol‐modifying properties, due to their reactivity with cysteine residues in proteins and with low‐molecular‐weight thiols [14, 17, 18, 19, 20]. Nevertheless, the chemical instability of acyclic thiosulfinates has imposed significant limitations on their utility in synthetic chemistry and biomedical applications [21]. In contrast, cyclic thiosulfinates (CTs), which are synthesized through the controlled oxidation of cyclic disulfides, have emerged as a structurally and functionally distinct subclass of organosulfur compounds [22]. Importantly, CTs demonstrate superior chemical stability compared to acyclic thiosulfinates, thereby facilitating their integration into diverse advanced scientific and technological applications.

This review aims to provide a detailed and critical overview of CTs, spanning (i) structural features and mechanistic insights into their reactivity with thiols, (ii) synthetic strategies for their preparation, and (iii) application in chemical biology and materials science. By integrating developments across these disciplines, we highlight how CTs have evolved from relatively obscure sulfur heterocycles to multifunctional platforms with broad translational potential.

## 2 Structural Features and Mechanistic Insights of Cyclic Thiosulfinates

CTs constitute a distinct class of sulfur‐containing heterocycles defined by the incorporation of a ─S(═O)─S─ functional motif within a conformationally constrained ring system. This structural element differentiates CTs from conventional cyclic disulfides by introducing bond polarization, stereochemical asymmetry, and increased conformational rigidity. Embedding the ─S(═O)─S─ unit within a cyclic framework further imposes ring strain and stereoelectronic bias, which are particularly significant in five‐ and six‐membered systems. These structural features modulate both thermodynamic stability and kinetic reactivity [22].

**FIGURE 1:**
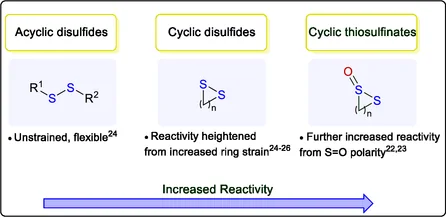
Schematic illustration highlighting the reactivity profiles of acyclic disulfides, cyclic disulfides, and cyclic thiosulfinates.

From an electronic perspective, the ─S(═O)─S─ linkage is intrinsically unsymmetrical. The strong inductive and dipolar effects of the S═O bond render the adjacent sulfur atom electrophilic, while promoting directional reactivity along the S─S axis. Additionally, computational and kinetic analyses have established ring size as a key determinant of reactivity, influencing both the activation barrier for thiolate attack and the thermodynamics of disulfide exchange [22, 23]. Notably, CTs exhibit significantly enhanced reactivity toward thiols relative to their parent cyclic disulfides, reflecting an interplay between ring strain and electronic activation (Figure 1) [22, 23]. In contrast, acyclic disulfides ge0nerally undergo slower thiol–disulfide exchange owing to the absence of ring strain [24]. Indeed, thiol–disulfide exchange has been reported to proceed up to 5000‐fold faster in cyclic disulfides than in their acyclic counterparts (Figure 1) [24, 25, 26]. Consistent with these observations, computational analyses of three‐ to eight‐membered cyclic disulfides and CTs revealed that the free energy of reaction (Δ*G*_rxn_) for thiolate nucleophilic attack is, on average, 1.6 kcal mol^−1^ lower for CTs than for the corresponding cyclic disulfides, further highlighting the enhanced thermodynamic favorability of thiol addition to CTs [23]. Although ring strain can accelerate desired thiol exchange reactions, excessive strain may reduce thermodynamic stability and increase susceptibility to ring opening, decomposition, or further oxidation. Thus, an optimal balance between stability and reactivity is often observed for CTs [22, 23]. Furthermore, thiol–disulfide exchange reactions are strongly influenced by pH because the reactive nucleophilic species is generally the thiolate anion rather than the neutral thiol [22, 23, 27]. Consequently, reaction rates increase with increasing thiolate concentration [22, 23]. In biological systems, the reactivity of CTs is further modulated by the surrounding redox environment and they have been shown to retain high cross‐linking efficiency under biologically relevant glutathione levels [22]. CT‐mediated cross‐linking has also been demonstrated to function as in vivo covalent stabilizers of mutant proteins [27].

**FIGURE 2:**
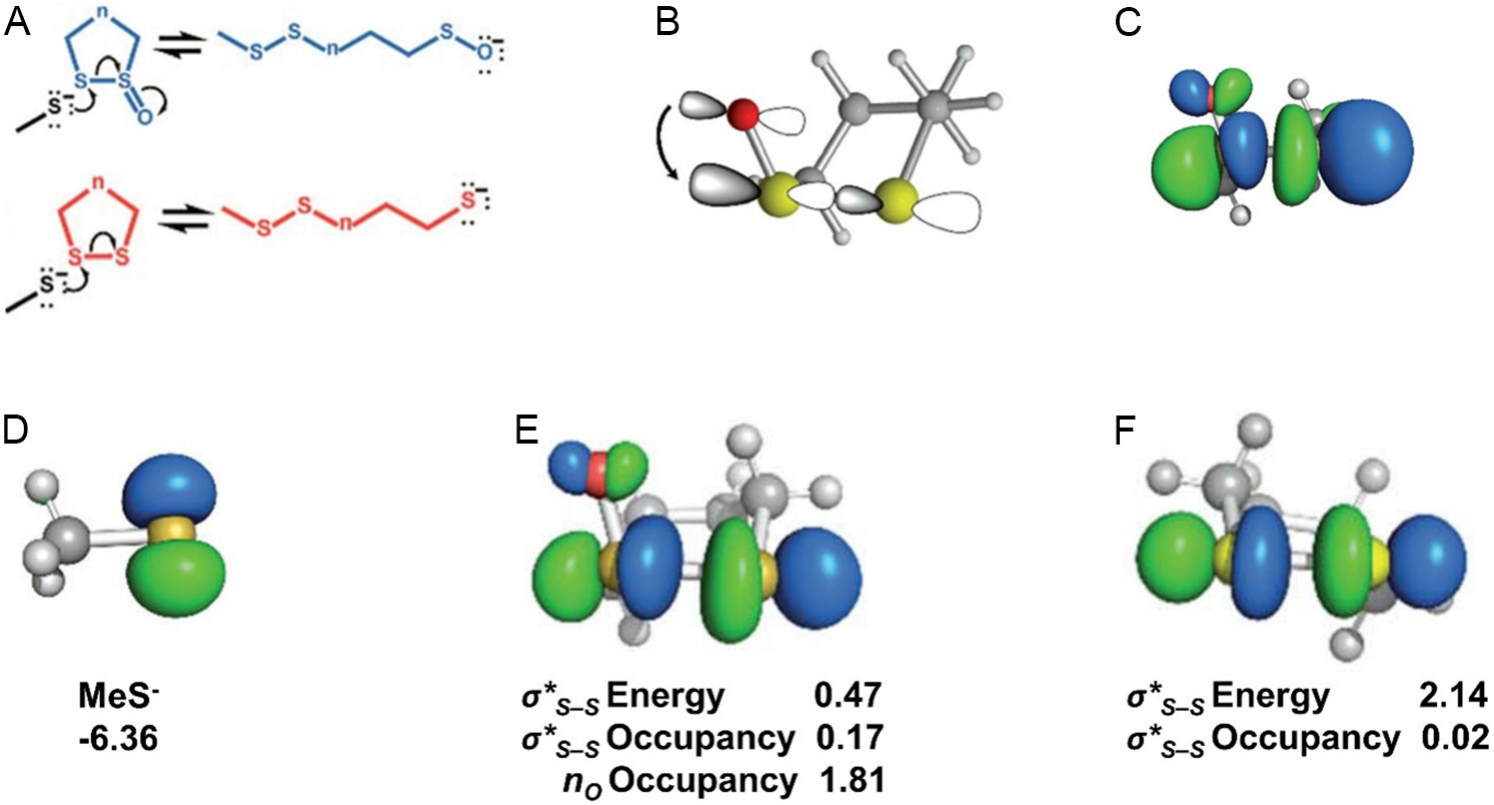
(A) Mechanism of thiol–disulfide exchange between nucleophilic methyl thiolate (MeS^−^) and a cyclic thiosulfinate (blue) or a cyclic disulfide (red). (B) Hyperconjugative *n*_O_ → σ*_S*─*S_ orbital interaction. (C) Computed LUMO of 6CT. (D) Visual representation of MeS^−^ HOMO and corresponding orbital energy. (E,F) Visual representation of σ*_S─S_ orbitals of 6CT (Figure 2E) and the six‐membered cyclic disulfides (Figure 2F), and the corresponding orbital energies and occupancies. Computed M06‐2X/6‐311++G(d,p) IEF‐PMC^H2O^. Energies are reported in eV. Adapted from Ref. [23]. Available under a CC‐BY‐NC 3.0 license. Copyright 2019, The Author(s).

A defining characteristic of CT reactivity is their propensity to undergo rapid thiol–disulfide exchange via a strain‐activated ring‐opening mechanism (Figure 2) [23]. Nucleophilic attack by a thiolate at the electrophilic sulfur center induces S─S bond cleavage, yielding a mixed disulfide and a transient sulfenic acid intermediate. Mechanistic investigations attribute this reactivity to the oxygen lone pair (*n*_O_) and σ* orbital of the S─S bond (σ*_S─S_) hyperconjugation, which weakens the S─S bond and lowers the activation energy for nucleophilic substitution. The σ*_S─S_ orbital energies of CTs are lower than those of cyclic disulfides, consistent with the *n*_O_ → σ*_S─S_ hyperconjugative interaction. As a representative example, 6CT and the corresponding six‐membered cyclic disulfide are shown in Figure 2. This interaction results in decreased *n*_O_ occupancy and increased σ*_S─S_ occupancy in CTs, supporting donor–acceptor orbital mixing. In contrast, cyclic disulfides show negligible σ*_S─S_ occupancy. The resulting lowering of the σ*_S─S_ orbital energy enhances frontier molecular orbital interactions with the MeS^−^ lone pair in the transition state, leading to accelerated nucleophilic substitution relative to cyclic disulfides [23]. These orbital interactions, reinforced by conformational constraints, underpins the “click‐like” kinetics observed in CT‐mediated thiol coupling reactions, enabling rapid and selective transformations under mild, aqueous conditions [22].

These distinct reactivity profiles have enabled diverse applications across chemical biology and materials science. In biological systems, CTs function as selective thiol cross‐linking reagents, preferentially targeting proximal cysteine residues while minimizing off‐target modification of isolated thiols. This selectivity has been leveraged for protein stabilization, covalent labeling, and the development of redox‐responsive therapeutics [27]. Additionally, CTs have been incorporated as conformationally constrained disulfide surrogates in bioactive molecules, offering enhanced stability and tunable reactivity [28, 29].

In materials chemistry, CTs have emerged as versatile building blocks for dynamic covalent networks. Their rapid and reversible thiol reactivity facilitates the formation of disulfide‐cross‐linked hydrogels with tunable mechanical properties and redox responsiveness [30, 31]. Furthermore, CT‐driven polymerization strategies have enabled the generation of phase‐separated soft materials, including coacervate systems that mimic biomolecular condensates [32].

Despite these advances, the broader utilization of CTs has been constrained by synthetic challenges arising from their intrinsic ring strain and oxidative lability. These factors often lead to competing ring‐opening or overoxidation pathways, necessitating carefully controlled, mild reaction conditions for their preparation and isolation.

## 3 Chemical Synthesis of Cyclic Thiosulfinates

Synthetic access to CTs has been predominantly achieved through the selective oxidation of cyclic disulfides. This strategy originated from early investigations of penicillin‐derived sulfur systems [33, ‍^34^] and was generalized across a broad range of ring sizes and substitution patterns. Central to this approach is the controlled mono‐oxidation of the disulfide linkage to generate the ─S(═O)─S─ motif while suppressing overoxidation to the corresponding thiosulfonate (─S(═O)_2_─S─). To this end, mild and chemoselective oxidants, including peracids (e.g., *m*‐CPBA) and hydrogen peroxide (H_2_O_2_), are typically employed under carefully tuned conditions to achieve high selectivity [22, 31, 35, 36].

This oxidative strategy has demonstrated broad functional group tolerance and has been successfully extended to structurally complex substrates, including cyclic peptides and natural product‐derived scaffolds [29]. Such compatibility underscores the utility of CTs for late‐stage functionalization, enabling their incorporation into multifunctional architectures relevant to chemical biology and materials science.

We have categorized CTs into three structural classes: (i) monocyclic CTs, (ii) bicyclic and tricyclic CTs, and (iii) macrocyclic CTs. Within this framework, the synthetic strategies reported in the literature for each class are discussed in the following sections.

### 3.1 Monocyclic Thiosulfinates

**SCHEME 1:**
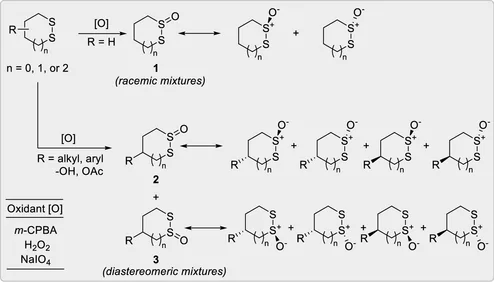
General reaction for the oxidation of cyclic disulfides to cyclic thiosulfinates (CTs) using oxidizing agents such as *m*‐CPBA, H_2_O_2_, or NaIO_4_. Unless otherwise noted, products **1** are obtained as racemic mixtures, whereas regioisomeric products **2** and **3** are obtained as mixtures of diastereomers. Throughout Schemes 2–7, structures will be designated according to the numbering shown in Scheme 1 (i.e., **1**, **2**, and **3**), with regiochemistry explicitly indicated and stereochemistry implied. Where a stereospecific outcome has been established, the corresponding stereochemistry will be explicitly depicted. The same nomenclature and stereochemical conventions are applied to the polycyclic (bicyclic and tricyclic) and macrocyclic CTs discussed in this review.

Monocyclic CTs represent the most extensively studied and synthetically accessible class, typically encompassing five‐ to seven‐membered ring systems. Their synthesis is predominantly achieved via the selective oxidation of the corresponding cyclic disulfides using mild oxidants such as *meta*‐chloroperbenzoic acid (*m*‐CPBA), hydrogen peroxide (H_2_O_2_), and sodium periodate (NaIO_4_) (Scheme 1) [22, 31, 37, 38, 39, 40, 41, 42, 43, 44]. A key advantage of monocyclic systems is their relatively straightforward precursor availability and predictable reactivity [22, 23].

The ring size has a pronounced influence on both the synthesis and reactivity of monocyclic CTs. Five‐ and six‐membered CTs possess greater ring strain, which enhances their reactivity toward nucleophiles through increased stereoelectronic activation of the S─S bond but can also reduce their stability, making them more susceptible to ring opening, disproportionation, or overoxidation [22, 23]. In contrast, seven‐ and eight‐membered CTs exhibit lower ring strain and are generally more stable, although the selective oxidation of the corresponding cyclic disulfides may require more carefully optimized reaction conditions to achieve complete conversion while minimizing overoxidation [22, 23]. Consequently, the balance between ring strain and conformational flexibility plays a critical role in determining both the synthetic accessibility and chemical reactivity of monocyclic CTs.

**SCHEME 2:**
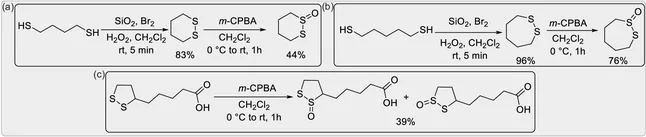
Synthesis of substituted five‐, six‐, and seven‐membered CTs using *m*‐CPBA.

Agar’s and Manetsch’s group reported the synthesis of six‐ and seven‐membered cyclic thiosulfinates (CTs) via oxidation of the corresponding cyclic disulfides using *m*‐chloroperbenzoic acid (Scheme 2) [22]. The requisite six‐ and seven‐membered cyclic disulfides were first obtained through intramolecular oxidative coupling of the corresponding dithiols (Scheme 2a,b). In addition, the oxidation of commercially available lipoic acid under similar conditions afforded a mixture of the corresponding regioisomeric CTs (Scheme 2c) [22].

**SCHEME 3:**
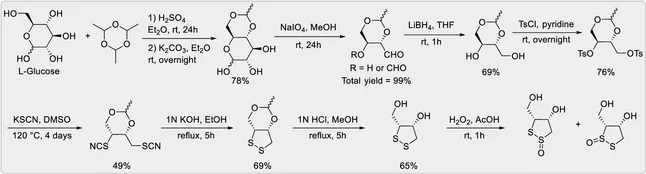
Synthesis of disubstituted five‐membered CTs using H_2_O_2_.

Hirai and coworkers reported the synthesis of disubstituted five‐membered CTs starting from *L*‐glucose (Scheme 3) [37]. Initial protection of the C‐4 and C‐6 hydroxyl groups of *L*‐glucose was followed by oxidative cleavage of the C‐2/C‐3 vicinal diol using sodium periodate, affording a mixture of the corresponding aldehyde and its formate derivative. Subsequent reduction of this mixture with lithium borohydride yielded the corresponding diol. Tosylation of the diol, followed by nucleophilic substitution with potassium thiocyanate, furnished the dithiocyanate intermediate. Alkaline hydrolysis of the thiocyanate groups, coupled with deprotection of the hydroxyl groups, provided the corresponding disubstituted cyclic disulfide. Final oxidation with hydrogen peroxide afforded a mixture of regioisomeric products.

**SCHEME 4:**
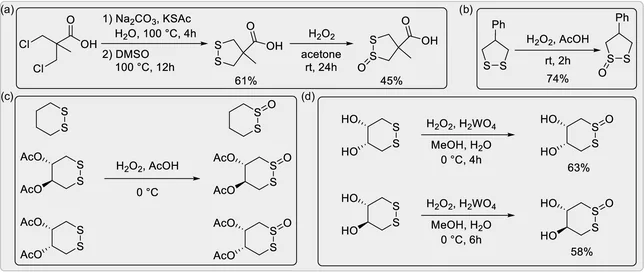
Synthesis of substituted five‐ and six‐membered CTs using H_2_O_2_.

Several other groups have reported the synthesis of substituted five‐ and six‐membered CTs via oxidation of the corresponding cyclic disulfides using hydrogen peroxide (Scheme 4). For instance, 4‐methyl‐1,2‐dithiolane‐4‐carboxylic acid 1‐oxide was synthesized in a three‐step sequence starting from commercially available 3,3‐dichloropivalic acid (Scheme 4a) [31]. The synthesis involved initial nucleophilic substitution with potassium thioacetate, followed by hydrolysis to generate the corresponding dithiol intermediate. Subsequent intramolecular oxidative coupling afforded the cyclic disulfide, which upon final oxidation with hydrogen peroxide furnished the target CT. In another example, 4‐phenyl‐1,‍2‐dithiolane 1‐oxide was obtained in 74% yield from the readily accessible cyclic disulfide 4‐phenyl‐1,2‐dithiolane (Scheme 4b) [38]. Similarly, unsubstituted and di‐*O*‐acylated six‐membered CTs were prepared from their corresponding cyclic disulfides using H_2_O_2_ in acetic acid (Scheme ‍4c) [39]. In contrast, the dihydroxy‐substituted CTs were synthesized using H_2_O_2_ in the presence of a catalytic amount of tungstic acid, affording the corresponding products as diastereomeric mixtures (Scheme 4d) [40].

Juaristi and Cruz‐Sánchez reported the synthesis of 4,4,5,5‐tetramethyl‐1,2‐dithiane mono‐*S*‐oxide in eight steps (Scheme ‍5) [41]. The sequence commenced with ethyl isobutyrate, which upon treatment with lithium diisopropylamide and chlorotrimethylsilane (TMSCl), generated the corresponding *O*‐silyl enol ether. Lewis acid‐mediated dimerization of this intermediate using titanium tetrachloride afforded a tetramethyl‐substituted succinate derivative. Subsequent reduction of the succinate, followed by tosylation and nucleophilic substitution with potassium thioacetate, furnished the di‐*S*‐acylated intermediate. Hydrolysis of the thioacetate groups and oxidative coupling with iodine yielded the corresponding 4,4,5,5‐tetramethyl‐1,2‐dithiane. Final oxidation with sodium periodate provided the target CT, 4,4,5,5‐tetramethyl‐1,2‐dithiane mono‐*S*‐oxide.

**SCHEME 5:**
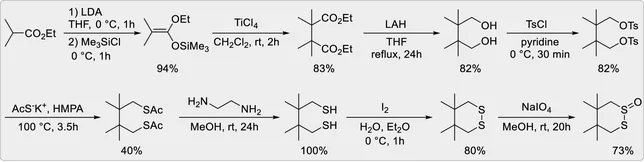
Synthesis of tetrasubstituted six‐membered CTs using NaIO_4_.

**SCHEME 6:**
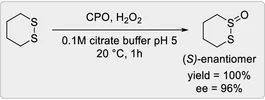
Synthesis of enantiopure six‐membered CT using chloroperoxidase and H_2_O_2_.

Boyd et al. reported the preparation of enantioenriched and enantiopure six‐membered CT via asymmetric enzymatic oxidation of the corresponding cyclic disulfides using chloroperoxidase (CPO) and hydrogen peroxide (H_2_O_2_) (Scheme 6) [42, 43]. This biocatalytic approach afforded the (*S*)‐enantiomer in quantitative yield with excellent enantiomeric excess.

**SCHEME 7:**
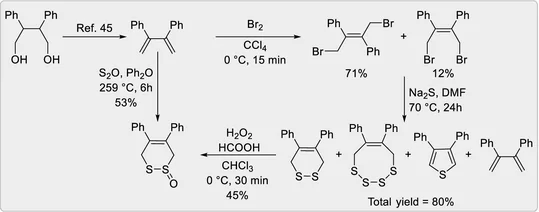
Synthesis of 4,5‐diphenyl‐3,6‐dihydro‐1,2‐dithiine 1‐oxide.

Dodson and coworkers reported the synthesis of the six‐membered CT, 4,5‐diphenyl‐3,6‐dihydro‐1,2‐dithiine 1‐oxide, via two distinct approaches (Scheme 7) [44]. In the first method, disulfur monoxide—generated in situ by pyrolysis of thiirane oxide—underwent a cycloaddition reaction with 2,3‐diphenylbutadiene (obtained by dehydration of 2,3‐diphenylbutane‐1,4‐diol) [45] to afford the target CT in 53% yield. In the second approach, 2,3‐diphenylbutadiene was subjected to bromination to give the corresponding dibromo intermediates, which upon treatment with sodium sulfide yielded a mixture of sulfur‐containing products, including the corresponding cyclic disulfide. Subsequent oxidation of this intermediate with hydrogen peroxide (H_2_O_2_) furnished 4,5‐diphenyl‐3,6‐dihydro‐1,2‐dithiine 1‐oxide.

### 3.2 Bicyclic and Tricyclic Thiosulfinates

Bicyclic and tricyclic thiosulfinates are structurally constrained cyclic sulfur‐containing compounds in which the thiosulfinate functional group is incorporated into fused, bridged, or spirocyclic ring systems. Compared with monocyclic analogues, these polycyclic architectures exhibit greater conformational rigidity and well‐defined 3D structures, which may influence their stereoelectronic properties and chemical reactivity [46, ‍^47^]. The synthesis of these compounds generally involves preparing the corresponding bicyclic or tricyclic disulfides, followed by carefully controlled, selective oxidation to the corresponding thiosulfinates, while minimizing overoxidation to higher sulfur oxides [33, 34, 48, 49, 50]. Owing to their conformationally constrained frameworks, bicyclic and tricyclic thiosulfinates are expected to display reactivity profiles that differ from those of monocyclic analogues. However, only a limited number of such compounds have been reported, and studies have primarily focused on their synthesis rather than their physicochemical properties, reactivity, or biological applications. Consequently, the structure–reactivity relationships of bicyclic and tricyclic thiosulfinates remain largely unexplored, highlighting the need for further investigation to fully realize their potential in chemical biology and materials science.

**SCHEME 8:**
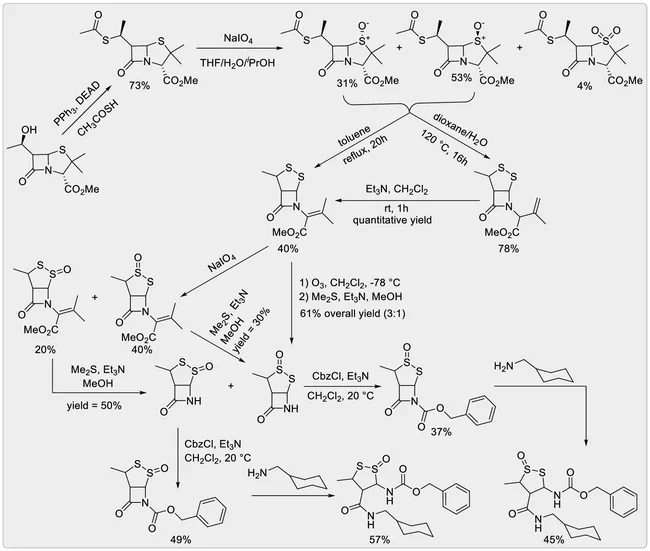
Synthesis of bicyclic thiosulfinates from penicillin sulfoxide.

Mak and Schulz reported the synthesis of bicyclic thiosulfinates derived from penicillin sulfoxide (Scheme 8) [33]. A hydroxyethyl‐substituted penicillin derivative was first prepared from readily available 6‐aminopenicillanic acid following a literature procedure [51, 52, 53]. This intermediate was converted to the corresponding acetylthioethyl sulfide in 73% yield under Mitsunobu/Volante conditions [54]. Subsequent oxidation with sodium periodate afforded a mixture of isomeric sulfoxides in 31% and 53% yields, along with a minor amount (4%) of the corresponding sulfone. Refluxing this mixture of sulfoxides in toluene yielded the bicyclic *β*‐lactam (bicyclic disulfide) in 40% yield. Improved yields were achieved by heating the sulfoxide mixture in dioxane in the presence of water to generate an isomeric intermediate, which, upon treatment with triethylamine, furnished the desired bicyclic *β*‐lactam more efficiently. Final oxidation with either sodium periodate or ozonolysis, followed by reductive workup, afforded the corresponding stable bicyclic thiosulfinates. Furthermore, the authors demonstrated the conversion of these bicyclic thiosulfinates into monocyclic thiosulfinates via a two‐step sequence: initial formation of the *N*‐Cbz‐protected derivative using benzyl chloroformate, followed by ring transformation upon treatment with cyclohexylmethylamine.

In a related context, earlier work reported by Perrone and coworkers [34] demonstrated that the oxidation of the 2‐thiacephem ring system (easily accessible using literature procedures) [55, 56, 57, 58] provides access to bicyclic thiosulfinates (Scheme ‍9), which serve as key intermediates in *β*‐lactam rearrangements. Controlled oxidation of penicillin‐derived substrates using *m*‐CPBA generated regioisomeric pairs of bicyclic thiosulfinates as the major products, while overoxidation afforded the corresponding thiosulfonates. Notably, these thiosulfinates and thiosulfonates underwent stereospecific sulfur dioxide extrusion under thermal or base‐mediated conditions to furnish (5*R*)‐penem derivatives with high stereochemical fidelity. This study established the critical role of sulfur oxidation state in dictating reactivity within constrained bicyclic systems and provided early mechanistic insight into bicyclic thiosulfinate‐mediated transformations. These findings complement the later work by Mak and Schulz (Scheme 8), in which the oxidation of penicillin‐derived intermediates similarly enabled access to stable bicyclic thiosulfinates, further underscoring the utility of *β*‐lactam frameworks as precursors to structurally complex and functionally relevant bicyclic thiosulfinate architectures.

**SCHEME 9:**
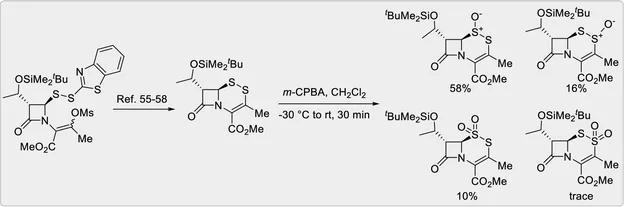
Synthesis of bicyclic thiosulfinates from the 2‐thiacephem ring system.

**SCHEME 10:**
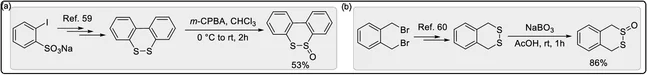
Synthesis of tricyclic and bicyclic thiosulfinates.

Boduszek and Kice reported the synthesis of a tricyclic thiosulfinate via oxidation of the corresponding tricyclic disulfide using *m*‐chloroperbenzoic acid (Scheme 10a) [48]. The requisite tricyclic disulfide precursor had been prepared previously from readily available starting materials [59]. Similarly, Singh and Field described the synthesis of bicyclic thiosulfinates through oxidation of the corresponding bicyclic disulfides using sodium perborate as the oxidant (Scheme 10b) [49]. The bicyclic disulfide substrates were obtained following established literature procedures [60].

Katritzky et al. developed a methodology for the interconversion of cyclic sulfinates into cyclic thiosulfinates. In this context, the bicyclic thiosulfinate 6‐dimethylamino‐3,3‐bis[(4‐dimethylamino)phenyl]−1,2‐benzodithiolane 1‐oxide was synthesized via activation of the corresponding cyclic sulfinate using thionyl chloride (SOCl_2_) in the presence of DMAP, followed by nucleophilic substitution with sodium hydrogen sulfide (NaSH), affording the target compound in 65% yield (Scheme ‍11) [50]. The requisite cyclic sulfinate precursor was prepared according to a previously reported procedure [61].

**SCHEME 11:**
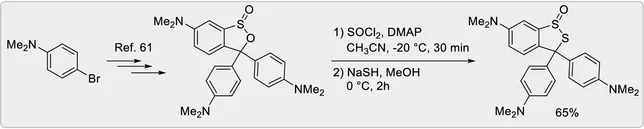
Synthesis of 6‐dimethylamino‐3,3‐bis[(4‐dimethylamino)phenyl]−1,2‐benzodithiolane 1‐oxide.

### 3.3 Macrocyclic Thiosulfinates

Macrocyclic thiosulfinates constitute a relatively recent and synthetically challenging class of cyclic thiosulfinates, typically derived from medium‐ to large‐sized cyclic disulfides (≥9‐‍membered rings). As with macrocycles in general, their synthesis is inherently challenging because of the unfavorable entropic cost of macrocyclization, together with the increased conformational flexibility and reduced ring strain of larger rings [47, 62, 63]. To date, the reported macrocyclic thiosulfinates are predominantly peptide‐ or peptidomimetic‐based systems. These compounds are generally prepared by solid‐phase or solution‐phase peptide synthesis to afford the corresponding dithiol precursors, followed by intramolecular disulfide formation and selective oxidation to generate the corresponding macrocyclic thiosulfinates [25, 26, 64, 65, 66]. Similar to tricyclic and bicylic thiosulfinates, only a limited number of macrocyclic thiosulfinates have been reported, with most studies focusing primarily on their synthesis. Although the applications of macrocyclic thiosulfinates remain largely unexplored, their conformational flexibility and dynamic sulfur chemistry suggest considerable potential for future applications in chemical biology and materials science.

**SCHEME 12:**
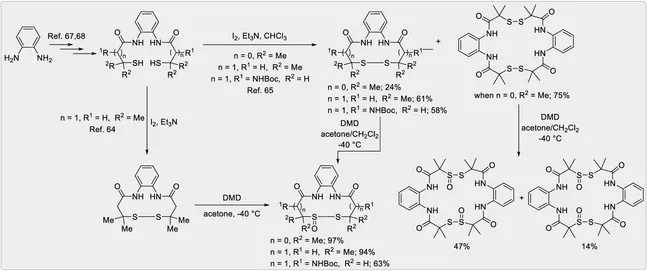
Synthesis of pseudopeptidic macrocyclic thiosulfinates.

The studies reported by Artaud and coworkers describe the synthesis and reactivity of pseudopeptidic macrocyclic thiosulfinates derived from the corresponding disulfide precursors (Scheme 12) [64, 65]. The requisite disulfide precursors were prepared following literature procedures [67, 68], and subsequent iodine‐mediated intramolecular oxidative coupling furnished the corresponding macrocyclic disulfides. Notably, for *n* = 0, a mixture of cyclic mono‐ and bis‐disulfides was obtained, which could be readily separated by crystallization. Controlled oxidation of the isolated cyclic disulfides using 2,2‐dimethyldioxirane (DMD) at low temperature afforded the corresponding pseudopeptidic macrocyclic thiosulfinates in good yields.

**SCHEME 13:**
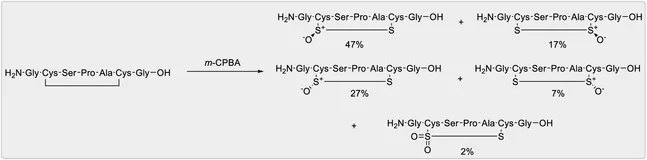
Synthesis of macrocyclic thiosulfinate peptide.

Lücke and coworkers investigated the conformational consequences of disulfide oxidation in cyclic peptides [25]. A model cyclic peptide was synthesized according to literature procedures [69] and subsequently oxidized using *m*‐chloroperbenzoic acid, a non‐regio‐ and non‐stereoselective oxidant. This transformation afforded a mixture of regio‐ and stereoisomeric macrocyclic thiosulfinates as the major products, along with minor amounts of the corresponding thiosulfonate (Scheme 13) [25], as confirmed by high‐performance liquid chromatography and nuclear magnetic resonance analysis. The resulting macrocyclic thiosulfinates were sufficiently stable for isolation and exhibited pronounced conformational changes due to incorporation of the sulfoxide functionality. These findings highlight the feasibility of introducing thiosulfinate motifs into macrocyclic peptide frameworks and underscore their utility in modulating peptide structure and reactivity.

**SCHEME 14:**
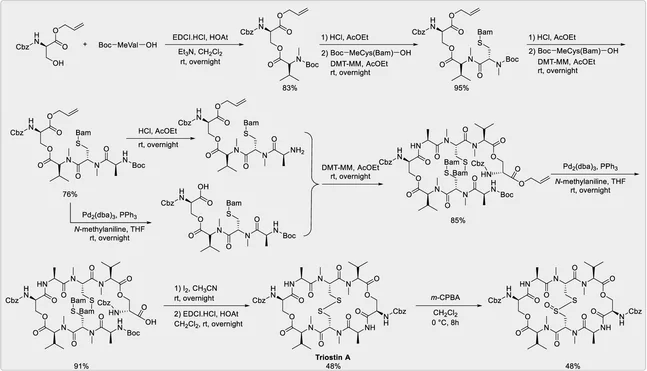
Synthesis of macrocyclic thiosulfinate analogue of triostin A.

A team led by Nagasawa reported the synthesis of macrocyclic thiosulfinates from the natural product triostin A via oxidation of the disulfide bridge using *m*‐chloroperbenzoic acid (Scheme 14) [26]. The parent macrocycle, triostin A, was prepared through a solution‐phase total synthesis involving stepwise assembly of the linear depsipeptide via coupling–deprotection sequences, followed by double macrocyclization and disulfide bond formation, delivering the target compound in 13 steps with an overall yield of ~17.5%. Subsequent oxidation of the disulfide linkage furnished the corresponding thiosulfinate analogue, demonstrating that structurally complex macrocycles can accommodate the ─S(═O)─S─ functionality without compromising structural integrity. Biologically, the thiosulfinate‐containing macrocycle retained activity; both triostin A and its oxidized analogue inhibited HIF‐1 DNA binding and suppressed HIF‐1α protein accumulation, exhibiting hypoxia‐selective cytotoxicity in MCF‐7 cells.

**SCHEME 15:**
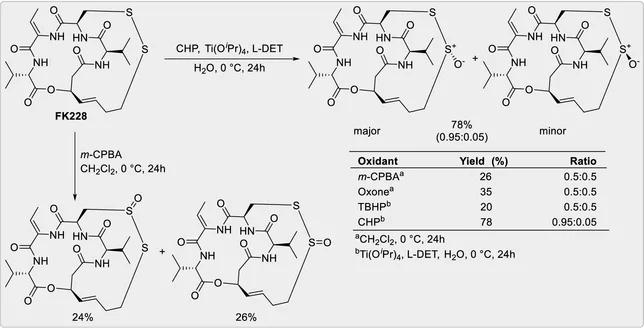
Synthesis of macrocyclic thiosulfinate analogue of FK228.

Sun et al. described an efficient and stereoselective method for the synthesis of macrocyclic thiosulfinates via oxidation of the corresponding disulfide linkage in FK228 (romidepsin) using cumene hydroperoxide (CHP) in the presence of a titanium‐based catalytic system (Scheme 15) [66]. FK228, a natural product featuring a bicyclic depsipeptide framework, has been synthesized previously through multiple total synthesis approaches [70, 71, 72, 73, 74]. In this study, selective oxidation of the disulfide bond was achieved using CHP in combination with Ti(*O*‐*i*‐Pr)_4_, L‐diethyl tartrate (L‐DET), and water, affording the corresponding thiosulfinate in good yield with high stereoselectivity under mild conditions. In contrast, conventional oxidants, such as *m*‐chloroperbenzoic acid, oxone, and *tert*‐butyl hydroperoxide (TBHP), produced mixtures of regio‐ and stereoisomeric macrocyclic thiosulfinates. Notably, the titanium‐based catalytic system enabled controlled mono‐oxidation of the disulfide bond while minimizing overoxidation to thiosulfonates, addressing a common challenge in thiosulfinate synthesis.

## 4 Application of Cyclic Thiosulfinates in Chemical Biology

CTs exhibit a unique combination of high reactivity and thiol selectivity, which has been elucidated through complementary experimental and computational studies. The development of CTs as thiol‐reactive reagents has progressed from fundamental chemical biology applications to validated therapeutic strategies.

**FIGURE 3:**
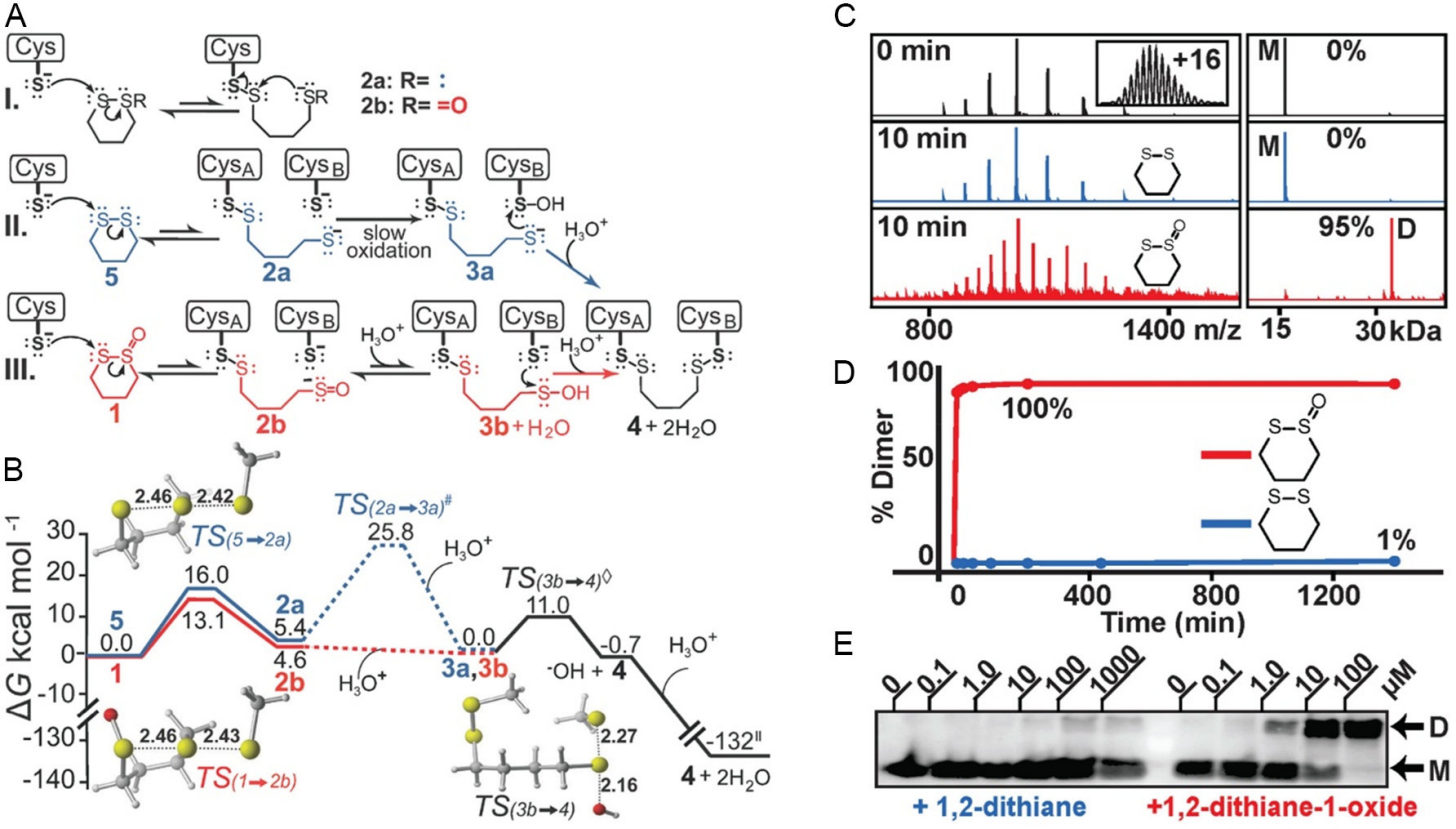
(A) Proposed mechanism of thiol cross‐linking using cyclic disulfides (blue) and cyclic thiosulfinates (red)—Formation of the first disulfide bond is reversible. Dead‐end modification is minimized by entropically favorable ring closure (I). Cross‐linking proceeds through condensation of Cys_A_, and a sulfenic acid derived from either rate‐limiting S‐oxidation of thiolate_B_ (II) or a cyclic thiosulfinate (III). (B) A potential energy surface for the nonenzymatic reaction of the proposed mechanism—The free energy values are computed using M06‐2X/6‐311+G(d,p) IEF‐PCM^H2O^//M06‐2X/6‐31+G(d,p) IEF‐PCM^H2O^. (C–E) Thiol cross‐linking by cyclic thiosulfinates kinetically stabilizes the SOD1 dimer in vitro and in cells—(C) Representative raw (Top left) and deconvoluted (Top right) mass spectra used for calculating cross‐linking rates (Middle). The 31,808 Da molecular mass of the cross‐linked dimer (D) supports the mechanism proposed. (D) After 10 min of incubation, 1,2‐dithiane‐1‐oxide and 1,2‐dithiane cross‐linked 95% and 0% of SOD1, respectively. (E) Western blot of superoxide dismutase 1 (SOD1) from HepG2 cells incubated with various concentrations of compounds for 30 min. Adapted with permission from Ref. [22]. Copyright 2018, American Chemical Society.

The foundational work by Agar and coworkers [22] established CTs as thiol‐selective cross‐linkers capable of targeting proximal cysteine residues while avoiding irreversible modification of isolated thiols (Figure 3). Using the cysteine pair in Cu/Zn superoxide dismutase (SOD1) as a model system, CTs have shown to undergo reversible thiol–disulfide exchange reactions that favor intramolecular disulfide bond formation rather than generating undesired “dead‐end” adducts. This selectivity arises from a strain‐activated ring‐opening mechanism that generates a sulfenic acid intermediate, enabling rapid disulfide bond formation. Consequently, CTs exhibit up to 10^4^‐fold faster reaction kinetics compared to cyclic disulfides. Density functional theory calculations attributed this rate acceleration to improved frontier molecular orbital interactions during thiolate attack and to the formation of a sulfenic acid intermediate after ring opening, which bypasses the otherwise rate‐limiting thiol oxidation step. The study further demonstrated that CTs retain high cross‐linking efficiency in the presence of biologically relevant concentrations of glutathione, are cell permeable, and function as effective intracellular cross‐linkers with low toxicity. Importantly, their ability to avoid dead‐end mono‐adduct formation positions CTs as click‐like reagents for thiol pairs, with utility in protein cross‐linking and bioconjugation.

**FIGURE 4:**
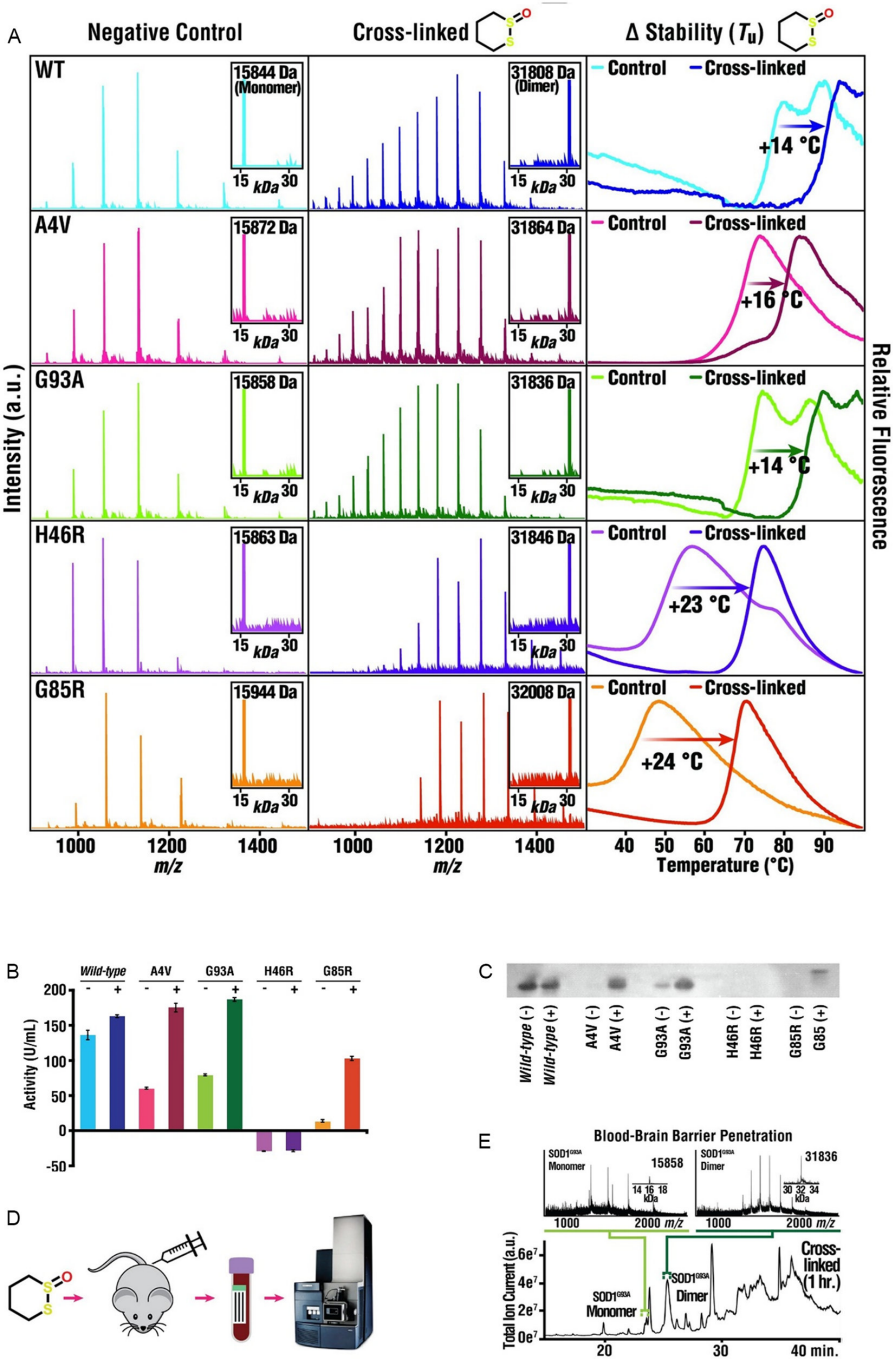
(A) *S*‐XL6‐mediated cross‐linking and stabilization of wild‐type and fALS SOD1 variants—Mass spectra of untreated (left column**,** average mass ± 1 Da) and *S*‐XL6 cross‐linked (middle column, average mass ± 2 Da) proteins are consistent with cross‐linking DSF results (right column), indicating that cross‐linking increased the thermal stability of SOD1 and its variants. (B,C) Enzymatic activity of SOD1 variants after *S*‐XL6 treatment—(B) Plate‐based biochemical and (C) gel‐based assays were used to assess the impact of cross‐linking on the enzymatic activity of SOD1 variants. Upon treatment with cross‐linker, the activity of SOD1^A4V^, SOD1^G93A^, and SOD1^G85R^ increased to that of wild‐type SOD1. Treatment with the cross‐linker did not affect the enzymatically inactive SOD1^H46R^. “+” and “−” denote samples treated with *S*‐XL6 and untreated samples, respectively. (D,E) *S*‐XL6 cross‐links SOD1 in an ALS mouse model and reduces aggregation in vitro—(D) Schematic of the pharmacodynamics workflow. (E) Target engagement via liquid chromatography–mass spectrometry analysis in transgenic (Tg) SOD1^G93A^ mouse brain dosed once at 30 mg/kg with *S*‐XL6 via subcutaneous injection. fALS, familial amyotrophic lateral sclerosis; SOD1, superoxide dismutase 1; DSF, differential scanning fluorimetry. Adapted from Ref. [27]. Available under a CC‐BY 4.0 license. Copyright 2024, The Author(s).

Building on this mechanistic and functional foundation, subsequent work from Agar’s group [27] translated CT chemistry into a disease‐relevant biological context (Figure 4). In particular, CTs were shown to function as in vivo covalent stabilizers of mutant proteins, using familial amyotrophic lateral sclerosis (fALS)‐associated SOD1 as a model system. Structure–activity optimization led to the identification of a 1,2‐dithiane‐1‐oxide (*S*‐XL6, also known as 6CT) that selectively cross‐links Cys_111_ residues at the SOD1 dimer interface. This modification conferred stabilization while preserving enzymatic activity, overcoming limitations associated with loss‐of‐function therapeutic strategies. Notably, *S*‐XL6 exhibited favorable pharmacokinetic properties, including oral bioavailability, low toxicity, blood–brain barrier penetration, and in vivo target engagement, resulting in increased protein half‐life in a mouse model. These findings provide strong proof of concept that CT‐mediated cross‐linking can stabilize disease‐associated proteins in vivo and represents a promising therapeutic approach for protein misfolding disorders such as amyotrophic lateral sclerosis (ALS).

Collectively, these studies delineate a clear progression in the field—from the identification of CTs as selective, rapid, and biocompatible thiol cross‐linking reagents to their emergence as precise covalent stabilizers in biological systems. This trajectory underscores the broader potential of CTs as a versatile platform for targeted protein stabilization, particularly in diseases driven by protein misfolding and instability.

## 5 Application of Cyclic Thiosulfinates in Materials Science

CTs have emerged as a versatile platform for biomaterials design, enabling the construction of dynamic, adaptive systems that span hydrogels, degradable networks, and phase‐separated soft matter. Across these applications, a unifying principle is the thiol‐triggered ring‐opening of CTs, which generates sulfenic acid intermediates and ultimately yields disulfide‐linked architectures with tunable reversibility.

**FIGURE 5:**
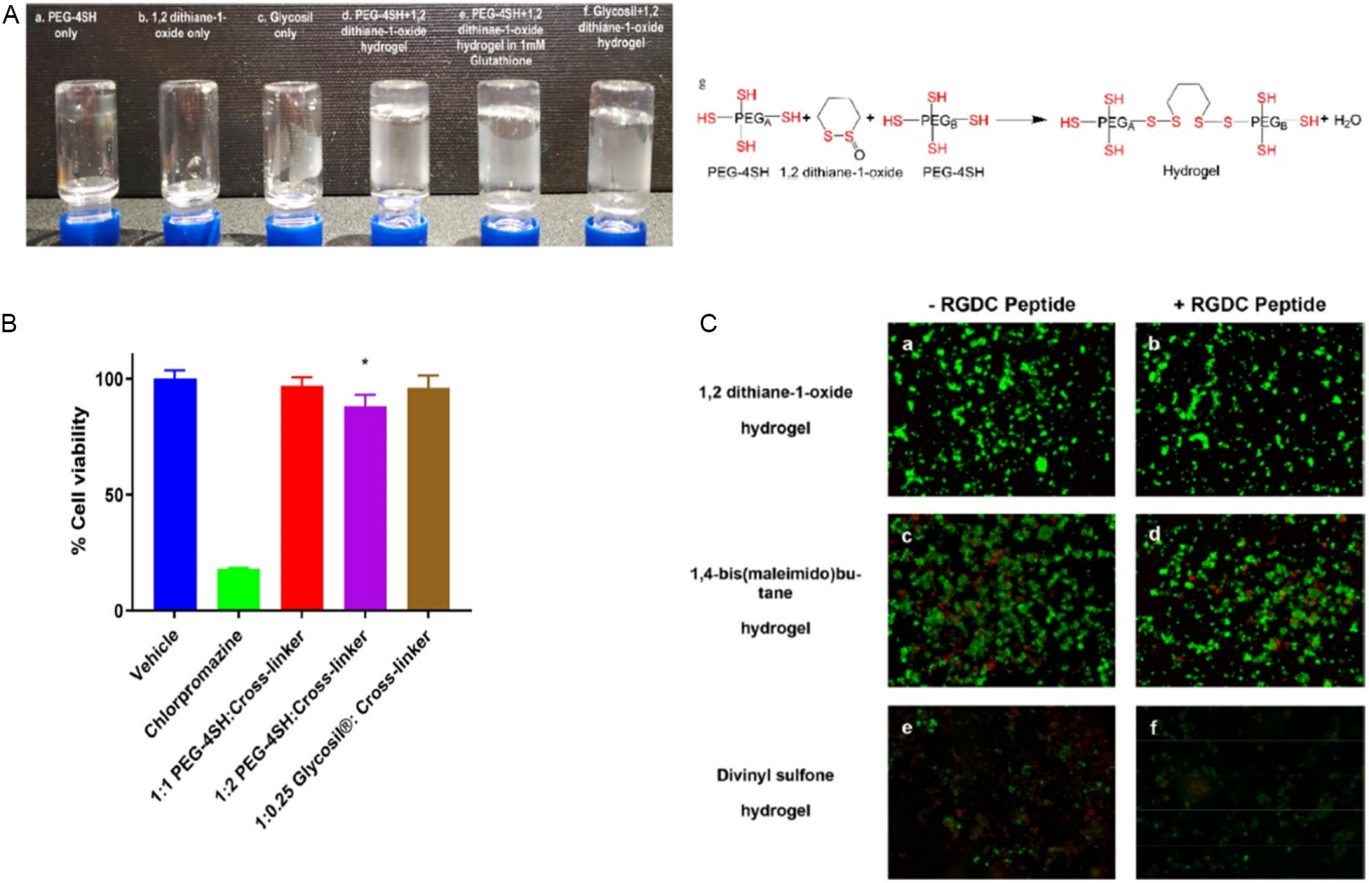
(A) Inverted test tubes demonstrating the formation of hydrogel (10% w/v) using PEG‐4SH and 1,2 dithiane‐1‐oxide—(a) PEG‐4SH‐only indicating no gelation; (b) 1,2‐dithiane‐1‐oxide‐only indicating no gelation; (c) Glycosil only showing no gelation; (d) gelation of PEG‐4SH and 1,2‐dithiane‐1‐oxide in PBS; (e) gelation of PEG‐4SH and 1,2‐dithiane‐1‐oxide in the presence of 1 mM glutathione; (f) gelation of Glycosil and 1,2‐dithiane‐1‐oxide; and (g) reaction of cross‐linking PEG‐4SH and 1,2‐dithiane‐1‐oxide to form the hydrogel. (B) Cell viability in the presence of hydrogel (10% w/v) cross‐linked using 1,2‐dithiane‐1‐oxide at 24 h. The cell viability was based on the metabolic activity of cells determined using ATPlite assay. No significant cytotoxicity was observed in the presence of hydrogel with 1:1 PEG‐4SH/cross‐linker (*p‐*value 0.59), while 1:2 PEG‐4SH/cross‐linker (indicated by “*”) showed 88% with statistical significance (*p‐*value 0.02). 500 μM of chlorpromazine was used as a positive control; (C) Live/dead cell assay of HepG2 cells 24 h post‐encapsulation—HepG2 cells are encapsulated during hydrogel synthesis by mixing PEG‐4SH with cross‐linkers in a 1:2 molar ratio (6.6% w/v). Cells encapsulated in PEG‐4SH‐1,2‐dithiane‐1‐oxide hydrogel exhibit the highest cell viability (a) with and (b) without addition of RGDC peptide. Cells encapsulated using 1,4‐bis(maleimido)butane (ca. 80‐fold lower cross‐linker concentration than 1,2‐dithiane‐1‐oxide) (c) with and (d) without RGDC peptide. Cells encapsulated using divinyl sulfone (ca. twofold lower cross‐linker concentration than 1,2‐dithiane‐1‐oxide) (e) with and (f) without RGDC peptide. Adapted with permission from Ref. [30]. Copyright 2021, American Chemical Society.

Early translation of CT chemistry into biomaterials was demonstrated by Agar and coworkers [30], wherein CTs were employed as thiol‐selective cross‐linkers for hydrogel formation (Figure 5). Reaction of 1,2‐dithiane‐1‐oxide (6CT) with thiol‐functionalized four‐arm polymers, including PEG‐thiol and hyaluronic acid–thiol, resulted in ultrafast gelation under physiological conditions, forming disulfide‐cross‐linked networks within seconds. These hydrogels exhibited redox‐responsive degradation in the presence of glutathione. The hydrogels enabled efficient protein encapsulation and controlled release, while maintaining low cytotoxicity and supporting the encapsulation, viability, and growth of HepG2 cells for up to 10 days. These findings established CTs as versatile, biocompatible, and redox‐responsive cross‐linkers for the fabrication of biodegradable hydrogels and tissue engineering materials.

**FIGURE 6:**
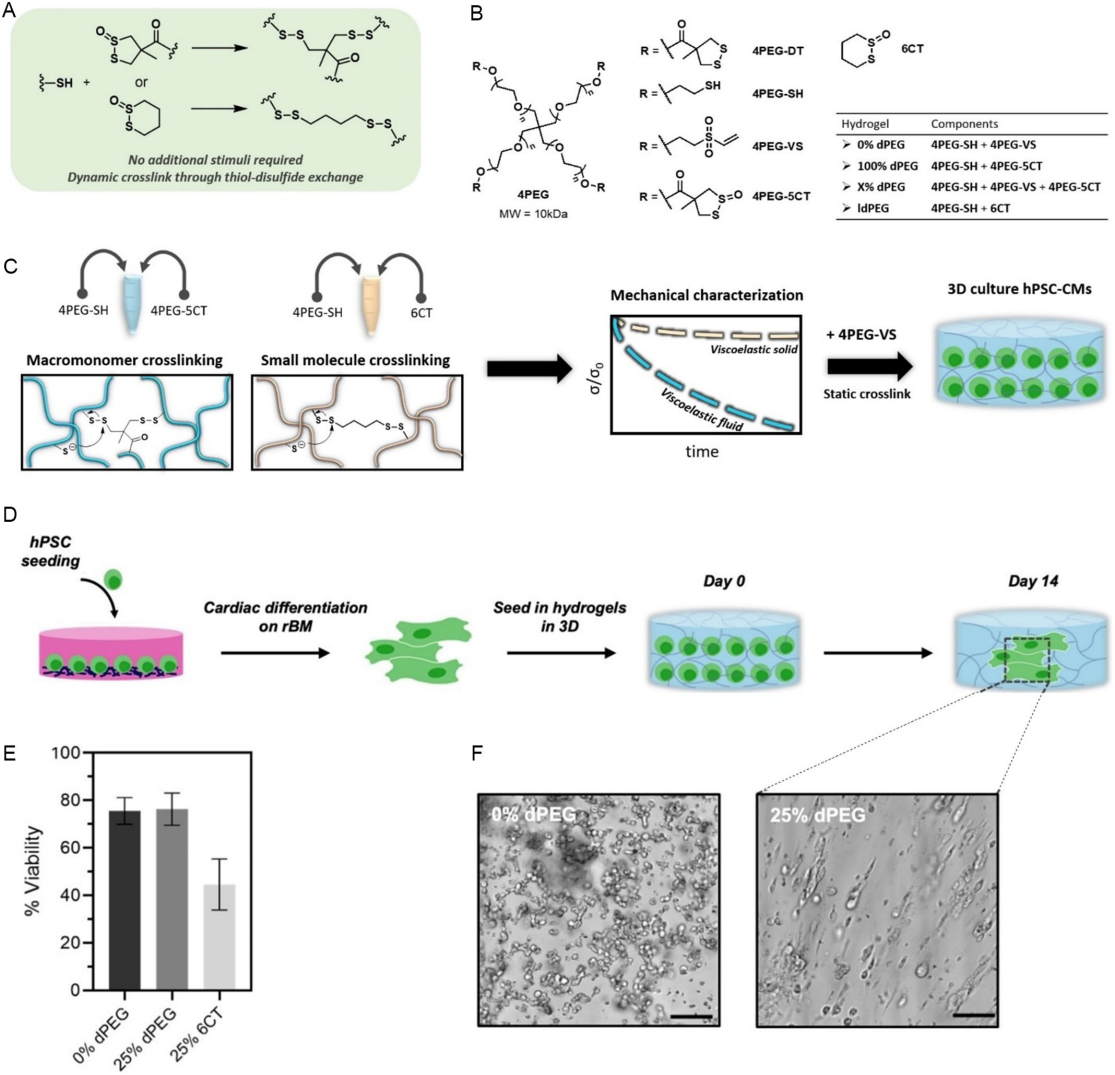
(A) Cyclic thiosulfinates cross‐linked using thiols without additional stimuli, yielding two disulfide bonds to form dynamic materials. (B) Components used and acronyms for disulfide‐cross‐linked hydrogels. (C) Application of the cyclic thiosulfinate cross‐linker on a macromolecule (4PEG‐5CT) or as a small molecule (6CT) leads to distinct stress relaxation profiles enabled by dynamic thiol–disulfide exchange in the hydrogel networks. These cross‐linking strategies are evaluated for the 3D culture of hPSC‐CMs that lack robust proliferation. (D) Schematic representation of hPSC cardiac differentiation, 3D encapsulation, and culture. (E) Cell viability percentages of human‐induced pluripotent stem cell‐derived cardiomyocytes after 24 h of 3D cell culture in 6 mM 0% dPEG, 25% dPEG, or 25% 6CT‐cross‐linked hydrogels. (F) Representative bright‐field images of human embryonic stem cell‐derived cardiomyocytes after a 14‐day culture in 0% dPEG and 25% dPEG hydrogels. Adapted from Ref. [31]. Available under a CC‐BY 4.0 license. Copyright 2023, The Author(s).

Building on this foundation, Kieltyka and coworkers [31] demonstrated that CT‐based cross‐links impart programmable viscoelasticity to hydrogel systems (Figure 6). Incorporation of mono‐*S*‐oxo‐4‐methyl asparagusic acid (5CT) into multiarm PEG networks enabled dynamic thiol–disulfide exchange, allowing precise tuning of stress relaxation behavior. By modulating thiol concentration and cross‐linking architecture, materials exhibiting transitions between fluid‐like and solid‐like mechanical responses were obtained, closely mimicking the extracellular matrix. Importantly, the materials supported the 3D culture of human pluripotent stem cell‐derived cardiomyocytes (hPSC‐CMs), promoting cell elongation and spontaneous beating, thereby demonstrating the utility of CTs as versatile cross‐linkers for engineering dynamic, biomimetic tissue culture matrices.

**FIGURE 7:**
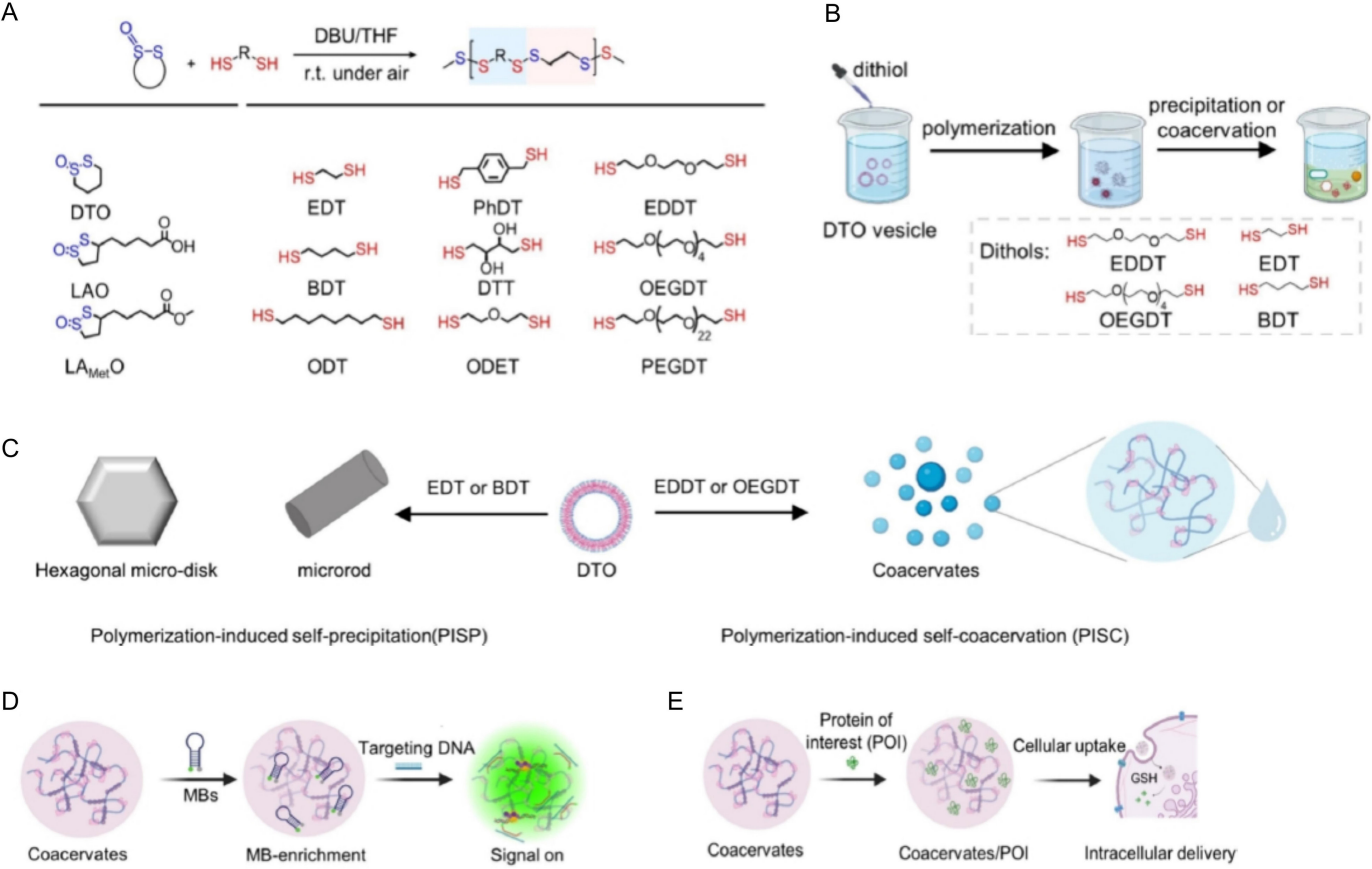
(A) Reaction equation of dithiols with cyclic thiosulfinates in THF catalyzed by DBU (0.01 equiv). The molar ratio of the dithiol and DTO was fixed to 1:2, and the polymerization was performed in THF at room temperature for 24 h. (B) Scheme for the DTO‐templated ROMPOC process. EDT, BDT, EDDT, and OEGDT were used as representative dithiols. (C) DTO templated PISP and PISC. ROMPOC of EDT(BDT) and DTO led to the formation of crystalline precipitates through PISP, whereas ROMPOC of EDDT(OEGDT) and DTO in situ formed microsized coacervates via PISC. (D) Schematic diagram of the P[(CRGGC/OEGDT)‐*a*‐DTO] coacervate in molecular beacon (MB)‐based DNA biosensing. (E) Schematic diagram of the coacervate in intracellular protein delivery. Adapted with permission from Ref. [32]. Copyright 2024, American Chemical Society.

More recently, the scope of CT‐based materials has expanded beyond cross‐linked networks to phase‐separated soft matter systems, as demonstrated by Mu et al. (Figure 7) [32]. In this work, 1,2‐dithiane‐1‐oxide (6CT, which is also abbreviated as DTO) served as a monomer in a ring‐opening, thiolate‐mediated polycondensation process, wherein the formation of sulfenic acid intermediates drove polymer growth. This process enabled polymerization‐induced self‐coacervation, leading to membraneless liquid droplets analogous to biological condensates. Furthermore, incorporation of cationic peptide motifs enhanced nucleic acid sequestration, highlighting the potential of CT‐derived poly(disulfides) as programmable biomaterials for DNA biosensing and protein delivery.

## 6 Summary and Outlook

CTs have emerged as a versatile and rapidly growing class of organosulfur heterocyclic compounds that possess a range of functional applications. Early studies established their formation via controlled oxidation of cyclic disulfides, revealing the structural consequences of introducing the ─S(═O)─S─ motif, including increased polarity, stereochemical asymmetry, and conformational bias. These features distinguish CTs from their disulfide counterparts and underpin their unique physicochemical properties.

Mechanistic investigations have provided a detailed understanding of CT reactivity, highlighting the combined roles of ring strain and *n*_O_ → σ*_S─S_ hyperconjugation in pre‐activating the S─S bond toward nucleophilic attack. This electronic and geometric activation enables rapid and selective reactions with thiols via a ring‐opening mechanism that generates disulfide products and sulfenic acid intermediates. The resulting click‐like reactivity, coupled with high chemoselectivity for proximal cysteine residues, has positioned CTs as powerful tools in chemical biology.

Building on these fundamental understandings, CTs have been applied across diverse fields, particularly in chemical biology and materials science. In biological systems, they serve as selective protein cross‐linkers, enabling covalent stabilization and modulation of structure and function. Concurrently, their dynamic thiol–disulfide chemistry has been leveraged in materials science to construct hydrogels, adaptive polymer networks, and phase‐separated soft materials with biomimetic properties.

Despite considerable progress, the chemistry of CTs is still emerging, with significant opportunities for further development. Expanding beyond disulfide oxidation as the primary synthetic strategy will be important to access structurally diverse CTs with improved control over regio‐ and stereoselectivity, as well as functional group compatibility. Such advances will facilitate the exploration of structure–reactivity relationships and broaden their applicability. Equally important is the ability to adjust CT reactivity and selectivity in complex biological environments. Strategies that integrate electronic tuning, ring strain modulation, and substituent design are expected to form CTs with optimized performance for specific applications.

Collectively, the convergence of improved synthetic accessibility, deeper mechanistic insight, and an expanding application landscape positions CTs as a promising platform at the interface of synthetic and medicinal chemistry, chemical biology, and materials science.

## Funding

This work did not receive any specific funding.

## Conflicts of Interest

The authors declare no conflicts of interest.
